# Disruption of p16 and Activation of Kras in Pancreas Increase Ductal Adenocarcinoma Formation and Metastasis in vivo

**DOI:** 10.18632/oncotarget.357

**Published:** 2011-11-23

**Authors:** Wanglong Qiu, Fikret Sahin, Christine A. Iacobuzio-Donahue, Dario Garcia-Carracedo, Wendy M. Wang, Chia-Yu Kuo, Doris Chen, Dan E. Arking, Andrew M. Lowy, Ralph H. Hruban, Helen E. Remotti, Gloria H. Su

**Affiliations:** ^1^ The Departments of Otolaryngology and Head and Neck Surgery, Columbia University College of Physicians and Surgeons, New York, NY 10032; ^2^ Department of Pathology, Columbia University College of Physicians and Surgeons, New York, NY 10032; ^3^ The Sol Goldman Pancreatic Cancer research Center, Department of Pathology, The Johns Hopkins University Medical Institutions, Baltimore, MD 21205; ^4^ Microbiology Department, School of Medicine, Ankara University, Turkey; ^5^ McKusick-Nathans Institute of Genetic Medicine, Johns Hopkins University School of Medicine, Baltimore, MD 21205; ^6^ Department of Surgery, Division of Surgical Oncology, Moores Cancer Center, University of California, San Diego, La Jolla, CA 92093-0987

**Keywords:** p16, LOH at Kras, pancreatic cancer, metastasis, mouse model, conditional knock-out, target therapy

## Abstract

Inactivation of tumor suppressor gene *p16/INK4A* and oncogenic activation of *KRAS* occur in almost all pancreatic cancers. To better understand the roles of *p16* in pancreatic tumorigenesis, we created a conditional *p16* knockout mouse line (*p16^flox/flox^*), in which *p16* is specifically disrupted in a tissue-specific manner without affecting *p19/ARF* expression. *p16^flox/flox^*; *LSL-Kras^G12D^*; *Pdx1-Cre* mice developed the full spectrum of pancreatic intraepithelial neoplasia (mPanIN) lesions, pancreatic ductal adenocarcinoma (PDA), and metastases were observed in all the mice. Here we report a mouse model that simulates human pancreatic tumorigenesis at both genetic and histologic levels and is ideal for studies of metastasis. During the progression from primary tumors to metastases, the wild-type allele of *Kras* was progressively lost (loss of heterozygosity at *Kras* or LOH at *Kras*) in *p16^flox/flox^*; *LSL- Kras^G12D^*; *Pdx1-Cre* mice. These observations suggest a role for *Kras* beyond tumor initiation. *In vitro* assays performed with cancer cell lines derived from primary pancreatic tumors of these mice showed that cancer cells with LOH at *Kras* exhibited more aggressive phenotypes than those retained the wild-type *Kras* allele, indicating that LOH at *Kras* can provide cancer cells functional growth advantages and promote metastasis. Increased LOH at *KRAS* was also observed in progression of human pancreatic primary tumors to metastases, again supporting a role for the *KRAS* gene in cancer metastasis. This finding has potential translational implications- future *KRAS* target therapies may need to consider targeting oncogenic KRAS specifically without inhibiting wild-type KRAS function.

## INTRODUCTION

Pancreatic cancer is the 5th leading cause of cancer deaths in the United States. The 5-year survival rate is only 5%, despite significant increases in our understanding of the molecular events that mediate its onset and progression [1]. More reliable early detection methods are needed, as well as increased understanding of the mechanisms of metastasis and therapeutic resistance [2].

*KRAS* is one of the most frequently activated oncogenes in human tumors, with 95% mutation frequency in pancreatic carcinomas [3, 4]. Point mutations in codon 12 of *KRAS* (*KRAS*^*G12D*^ or *KRAS*^*G12V*^) are the most commonly identified in human pancreatic adenocarcinoma. This and other mutations that activate *KRAS* have been found in the earliest stages of pancreatic intraepithelial neoplasia (PanIN)-a precursor to infiltrating pancreatic ductal adenocarcinoma (PDA). PanIN can be divided into different grades (1A, 1B, 2 and 3) according to the degree of architectural and cytological atypia [5]. This association between *KRAS* mutations and PanIN, along with data from *in vitro* transformation assays, support the concept that pancreatic tumorigenesis can be initiated by activated KRAS.

Cellular responses to Ras activation vary and depend on the cell type, Ras isoform, expression level, and wild-type allele status [6, 7]. For example, targeted overexpression of activated Kras in the pancreas often leads to the development of pancreatic acinar hyperplasia or dysplasia, but not invasive cancer [8, 9]. Conversely, expression of activated *Kras*^*G12D*^ at a physiological level (in *LSL-Kras*^*G12D*^ mice), induced by *Pdx1-Cre* or *p48-Cre*, is sufficient to initiate the development of murine PanIN, some of which progress to PDA after a period of latency [10, 11]. The oncogenic function of mutant Kras protein is therefore significantly influenced by the expression level of the mutant *Kras* allele or the ratio of the wild-type to mutant Kras protein. Increasing evidence supports the latter hypothesis that the wild-type *Kras* allele can also impact cellular responses [7, 12, 13].

A human PDA sample contains an average of 63 genetic alterations [14]. Among them, *p16* (*INK4A*) is inactivation in virtually all human PDA [15]. The molecular mechanisms of *p16* inactivation include promoter methylation, missense mutation, and small deletion accompanied by loss of heterozygosity (LOH) [15, 16]. Other than small deletions, methylation and missense mutations specifically targeted *p16* and did not involve *p14* (*ARF*), another tumor-suppressor gene with distinctive protein structure and function but shares coding exons with *p16* [15-20]. In mice, activation of *Kras* alone is sufficient to induce the development of preinvasive lesions [11], and the deletion of the *p16/19* (*INK4A/ARF*) locus rapidly advances progression to invasive cancer as observed in *LSL- Kras*^*G12D*^*; p16/19*^*−/−*^*; Pdx1-Cre* mice [18, 21]. However, the role of *p16* in pancreatic tumorigenesis cannot be separated from that of *p19* in this model [18, 21]. *p16*^*−/−*^ mice (which retain *p19/ARF*) spontaneously develop and die from a variety of malignant tumors, including soft-tissue sarcoma, lymphoma, and melanoma [22], so the potential tumorigenic effects of *p16* loss in the pancreas are not known. To investigate the role of *p16* in pancreatic tumorigenesis, the most frequently mutated gene in human PDA, we generated conditional *p16* knockout mice. In these mice only exon1α of the *p16/19* locus is flanked by loxP sites, to inactivate only the *p16* allele in a tissue-specific manner without affecting the expression of p19.

## RESULTS

### Targeted deletion of *p16* in the pancreas does not affect development of pancreatic cell lineages

**Figure 1:**
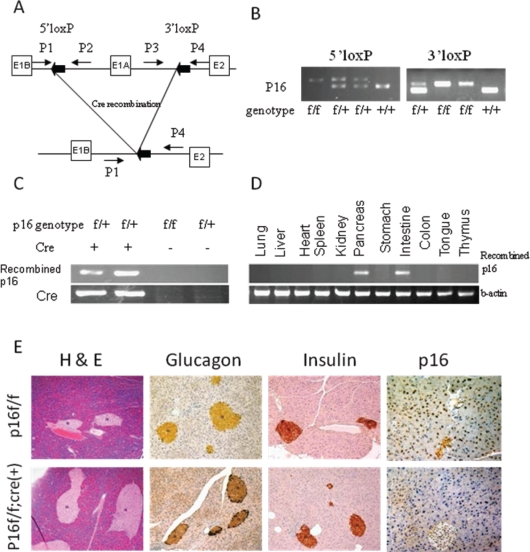
Tissue-specific inactivation of *p16* in mice (A) Schematic of the PCR strategies for genotyping and detecting Cre-mediated recombination. (B) The wild-type (*p16*^*+/+*^), *p16*^*flox/+*^, and *p16*^*flox/flox*^ genotypes were identified by PCR. (C) The recombined fragment of *p16* was detected only in mice with Cre transgene expression, and in the pancreas and intestine, but not in the other tissues of adult *p16*^*flox/flox*^; *Pdx1-Cre* mice (D). (E) Histological and immunohistochemical analyses of pancreatic sections from *p16*^*flox/flox*^; *Pdx1-Cre* mice showed normal development of pancreatic cell lineages with significant reduction of p16 protein expression.

In *p16*^*flox/flox*^ mice, exon1α of both alleles of *p16* alleles are flanked by loxP sites (Fig. 1). Mutant *p16*^*flox/flox*^; *Pdx1-Cre* mice were born at normal frequency and had no evidence of gross anatomic or physiological abnormalities. In adult *p16*^*flox/flox*^; *Pdx1-Cre* mice, tissue-specific genomic recombination of *p16* was restricted to the pancreas and intestine (Fig. 1D). The expression levels of amylase, insulin, and glucagon in the mutant mice appeared normal (Fig. 1E and data not shown). Immunohistochemical analysis demonstrated the loss of the p16 nuclear labeling in all three pancreatic cell lineages in the *p16* ^*flox/flox*^*; Pdx1-Cre* mice, while diffuse nuclear labeling for p16 was observed in the pancreas of the *p16* ^*flox/flox*^ control mice (Fig. 1E). The mutant and control mice had similar responses to the glucose tolerance test (data not shown). Finally, none of the *p16*^*flox/flox*^; *Pdx1-Cre* mice under 18 months of age (n=16) developed pancreatic neoplasms, therefore *p16* inactivation alone is not sufficient to initiate pancreatic tumorigenesis.

### Combined *p16* inactivation and *Kras* activation promote pancreatic tumor progression and metastasis

**Figure 2:**
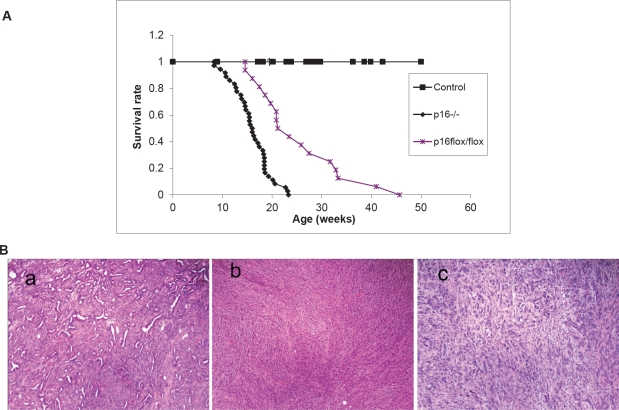
Effects of *p16* inactivation and Kras activation on survival and pancreatic tumor development (A) The median survival time was 15.5± 3.8 weeks (n = 36) for *p16*^*−/−*^; *LSL-Kras*^*G12D*^; *Pdx1-Cre* mice and 25.5± 8.9 weeks (n = 16) for *p16*^*flox/flox*^; *LSL-Kras*^*G12D*^; *Pdx1-Cre* mice. The control mice were mixed genotypes without the *LSL- Kras*^*G12D*^ allele; n = 115. (B) The most common histologic variant in the *p16*^*−/−*^; *LSL-Kras*^*G12D*^; *Pdx1-Cre* and *p16*^*flox/flox*^; *LSL-Kras*^*G12D*^; *Pdx1-Cre* mouse models was comprised predominantly of malignant glands characterizing as well to moderately differentiated adenocarcinoma (a) with a minor component composed of more poorly differentiated components of sarcomatoid (b) and undifferentiated anaplastic (c) carcinoma. Shown here are representative histology from *p16*^*−/−*^; *LSL-Kras*^*G12D*^; *Pdx1-Cre* mice.

To explore the dynamics of combined *p16* biallelic inactivation and *Kras* activation in pancreatic tumor development and progression in the setting of intact *p19, p16*^*−/−*^; *LSL- Kras*^*G12D*^; *Pdx1-Cre* mice and *p16*^*flox/flox*^; *LSL- Kras*^*G12D*^; *Pdx1-Cre* mice were generated and characterized. *p16*^*−/−*^; *LSL- Kras*^*G12D*^; *Pdx1-Cre* mice and *p16*^*flox/flox*^; *LSL- Kras*^*G12D*^; *Pdx1-Cre* mice had dramatically reduced median survival time (15.5± 3.8 weeks, n=36, and 25.5± 8.9 weeks, n=16, respectively) compared with *p16*^*+/+*^; *LSL- Kras*^*G12D*^; *Pdx1-Cre* mice (~15 months) [11] (Fig. 2A).

Other than the differences in median survival, the *p16*^*−/−*^; *LSL- Kras*^*G12D*^; *Pdx1-Cre* and *p16*^*flox/flox*^; *LSL- Kras*^*G12D*^; *Pdx1-Cre* mice were very similar in their tumor development and metastasis patterns. Mice of the *p16*^*−/−*^; *LSL- Kras*^*G12D*^; *Pdx1-Cre* genotype were normal until 6 weeks of age. Between 6 and 24 weeks of age, they started to exhibit weight loss, jaundice, ascites, and increased abdominal girth (Fig. S2). A series of autopsies revealed the presence of solid pancreatic tumors ranging from 3 to 30 mm in diameter. The tumors were grossly firm with irregular and ill-defined margins, frequently adherent to adjacent organs (Fig. S2). Multiple tumor nodules were often visible, indicating that neoplasms of this genotype were multifocal. Histological analysis revealed a glandular pattern of well-differentiated to moderately differentiated pancreatic adenocarcinomas resembling human PDA was the predominant histologic component observed in both mouse models. Adenocarcinomas were identified in 21of 22 invasive carcinomas in *p16*^*−/−*^; *LSL- Kras*^*G12D*^; *Pdx1-Cre* mice and comprised ≥ 50% in 13 of the 21 invasive carcinomas (Fig. 2B-a, Table S1) and in 9 of 9 in *p16*^*flox/flox*^; *LSL- Kras*^*G12D*^; *Pdx1-Cre* mice and comprised ≥ 50% in 5 of the 9 in *p16*^*flox/flox*^; *LSL- Kras*^*G12D*^; *Pdx1-Cre* mice (Fig. S3f, Table S2). Undifferentiated spindle cell and undifferentiated anaplastic components were also observed in most carcinomas although at low percentages (Fig. 2B, S3, Tables S1, S2). The varied morphologic spectrum of tumor phenotypes and heterogeneity within individual tumors is a common feature of these models and might result from the multifocal nature of the carcinomas.

**Figure 3:**
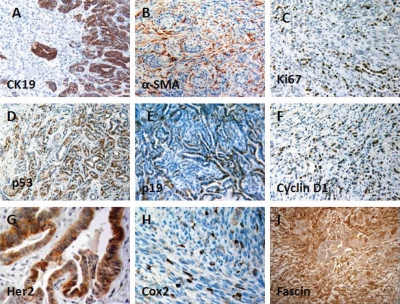
Immunohistochemical features of PDA in *p16^flox/flox^*; *LSL-Kras^G12D^*; *Pdx1-Cre* mice (A) CK19 immunolabeling was strong and diffuse in well-differentiated tumor, but negligible in the poorly differentiated or undifferentiated components; (B) Myofibroblasts, identified by α-SMA immunostaining, were associated with a desmoplastic stromal response and were commonly associated with invasive pancreatic cancer; (C) PDA cells showed increased proliferation, compared with normal tissue, based on increased nuclear staining of Ki-67; Nuclear expressions of p53 (D) and p19 (E) were detected in PDA. Cyclin D1 (F), Her-2 (G), and Cox-2 (H), and Fascin (I) were also overexpressed in PDA.

Invasive pancreatic adenocarcinomas expressed high levels of CK19 in well-differentiated regions and lower levels in sarcomatoid regions (Fig. 3A), whereas anaplastic regions showed no reactivity with anti-CK19. These anaplastic undifferentiated tumors had high mitotic activity, marked nuclear atypia, and dramatic cellular pleomorphism. The invasive carcinomas were usually associated with a desmoplastic stromal response, as shown by positive α-SMA labeling of myofibroblasts (Fig. 3B). Ki67 immunoreactivity, which indicates cell proliferation, was significantly increased in PDA compared with normal pancreatic tissue (Fig. 3C). Increased p53 and p19 expressions were most frequently detected at the tumor margin, transit areas, and in well-differentiated PDA (Fig. 3D, E). Cyclin D1 and Fascin were also highly expressed by PDA (Fig. 3F, I). Immunoreactivity for Her2 and Cox-2 varied; they were restricted to a subset of PDA and undifferentiated sarcomatoid carcinoma (Fig. 3G, H). Immmunohistochemistry analyses were also performed on precancerous lesions. Increasing levels of immunoreactivity to Fascin, Ki67, Her-2, Cyclin D1, Cox-2 were associated with mPanIN progression to invasive cancer as they are in humans (Table 1) [23], which further validated the similarities between the invasive pancreatic tumors found in our models and human PDA.

**Table 1:** Immunohistochemical profile of the precancerous lesions and pancreatic ductal adenocarcinoma of *p16^flox/flox^*; *LSL-Kras^G12D^*; *Pdx1-Cre* mice

Marker	Normal	PanIN-1*	PanIN-2	PanIN-3	PDA*
**Fascin**	−	+/−	++	++	+++
**Ki67**	+/−	+/−	+	++	++
**Her-2**	−	−	+	++	++
**Cyclin D1**	−	+/−	+	++	+++
**Cox-2**	−	−	+	++	++

* PanIN, pancreatic intraepithelial neoplasia; PDA, pancreatic ductal adenocarcinoma

The invasive carcinomas that developed in *p16*^*−/−*^; *LSL- Kras*^*G12D*^; *Pdx1-Cre* mice involved extensive amounts of peripancreatic tissue and frequently formed metastases. Numbers of metastases increased with age but were not restricted to older mice. Table S1 is a summary of *p16*^*−/−*^; *LSL- Kras*^*G12D*^; *Pdx1-Cre* mutant mice that had been sacrificed by age for histology analyses. Metastases were identified in 44% of the mutant mice (7/16, Table S1) that were <4 months-old, 69% of those >4 months (9/13). When mutant mice were sacrificed because they had reached the non-thriving stage, 100% had metastases (n=14). Almost all of the non-thriving animals had overly distended abdomens, resulting from the accumulation of hemorrhagic ascites (Fig. S2). The distribution pattern of the metastases is similar to that of human disease. Most metastases involved the liver and regional lymph nodes (Fig. S4, Table S1), with direct local invasion of the duodenum, stomach, diaphragm, and spleen. Metastases to lymph nodes, liver, lung, and testis were also observed (Fig. S4). The mice frequently had obstructions of the bile duct or small bowel (Fig. S2). The liver metastases most frequently had a glandular morphology, with less frequent undifferentiated sarcomatoid and anaplastic components (Fig. S4). Therefore, not only the invasive pancreatic tumors observed in our models mimic human PDA, here we also report that the combined *p16* biallelic inactivation and *Kras* activation can promote frequent and consistent metastases that mimic the distribution pattern of metastases observed in advanced human PDA.

### Inactivation of *p16* did not alter the histologic presentation of mPanIN initiated by oncogenic *Kras*

**Figure 4:**
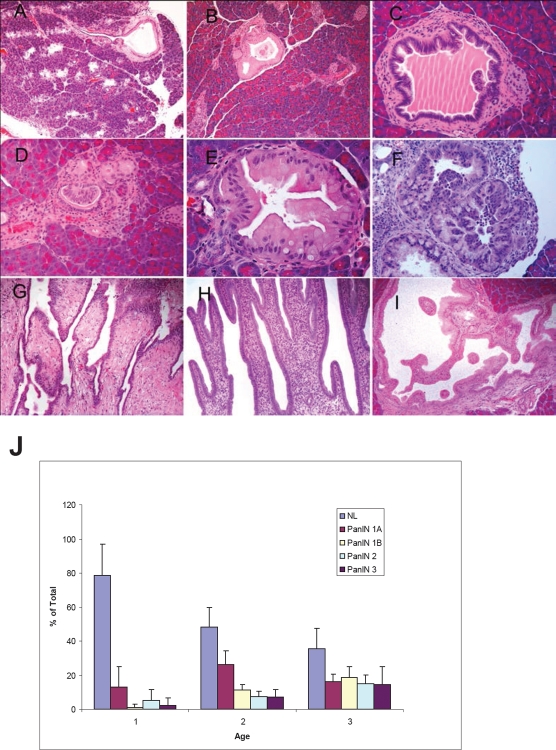
mPanIN progression in the pancreata of *p16^−/−^*; *LSL-Kras^G12D^*; *Pdx1-Cre* mice (A) An mPanIN lesion might narrow or obstruct the interlobular pancreatic duct (arrow); (B) mPanIN-1A with mild atypia (gastric foveolar type epithelium); (C) low-grade mPanIN-1B to mPanIN-2 lesion with mild cytologic atypia with focal micropapillary architecture; (D) focal mPanIN-2 lesion associated with background PanIN-1 lesion and (E) mPanIN-2 lesion with moderate cytologic atypia with focal micropapillary architecture; (F) High-grade mPanIN-3 lesion displays complete loss of cellular polarity, significant nuclear atypia, and budding of cell clusters into the ductal lumen. In addition to invasive tumors, pre-invasive occasional intraductal lesions with focal papillary (G, H) and cystic dilation (I), and multifocal mPanIN lesions with varying degrees of dysplasia were identified. (J) Histologic assessment of mPanIN progression in *p16*^*−/−*^*; LSL- Kras*^*G12D*^*; Pdx1-Cre* mice. To quantify mPanIN progression, the total number of ducts, including normal and various stages of mPanINs, was scored in 3 age groups of *p16*^*−/−*^*; LSL- Kras*^*G12D*^*; Pdx1-Cre* mice. Percentages (±SEM) of normal (NL) and neoplastic ducts by grade (mPanIN-1A, mPanIN-1B, mPanIN-2, and mPanIN-3) in the three groups are presented here. The average age for each group is cohort 1, 1.1 months (n=8); cohort 2, 2.9 months (n=5); and cohort 3, 4 months (n=5).

Tumors from *p16*^*−/−*^; *LSL- Kras*^*G12D*^; *Pdx1-Cre* mice were collected from mice at 4–6, 11–13 and 15–17 weeks and histologic analyses were performed. A full spectrum of mPanIN lesions were observed in the *p16*^*−/−*^*; LSL- Kras*^*G12D*^*; Pdx1-Cre* mice (Fig. 4 B–F), indicating that the biallelic inactivation of *p16* accelerated but did not alter the pathological progression of the *LSL- Kras*^*G12D*^*; Pdx1-Cre* mice [11]. At ~1 month of age, 5/8 *p16*^*−/−*^*; LSL- Kras*^*G12D*^*; Pdx1-Cre* mice had focal mPanIN lesions (predominantly low grade) but no PDA. The remaining 3 had normal pancreatic histology (Fig. 4J). At 3-4 months, *p16*^*−/−*^*; LSL- Kras*^*G12D*^*; Pdx1-Cre* mice had a significantly increased overall number and advanced grade of mPanINs compared with 1-month old mice (Fig. 4J). Mutant mice that were >3 months old all had multifocal PDA. High-grade mPanIN-3 lesions were frequently associated with PDA. The control littermates had normal pancreatic histology at each time point. Infrequently, focal and noninvasive cystic papillary neoplasms, resembling human intraductal papillary mucinous neoplasms were also observed (Fig. 4G–I) [24].

Similar histological progression pattern from precancerous lesions to invasive cancer and metastasis was also observed in the *p16*^*flox/flox*^; *LSL- Kras*^*G12D*^; *Pdx1-Cre* mice (Fig. S3), again demonstrating that p16 inactivation promotes progression but does not alter tumor initiation and histology.

### Loss of the wild-type *Kras* allele promotes pancreatic tumor cell proliferation and metastasis in mice and humans

Primary cell cultures were established from 12 primary tumors and 2 metastases of the *p16*^*flox/flox*^; *LSL- Kras*^*G12D*^; *Pdx1-Cre* mice (Two of the 12 primary tumors and the two metastases were harvested from the same mice). From these primary cell cultures, we further established 464 clonal cancer cell lines from the 12 pancreatic primary tumors and 42 clonal cancer cell lines from the metastasis. Smad4 and p19 proteins were detected in all the clonal cancer cell lines examined (Fig. S5). We sequenced *p53* cDNA of ~100 clonal cancer cell lines but did not uncover any *p53* mutation in these cells.

**Figure 5:**
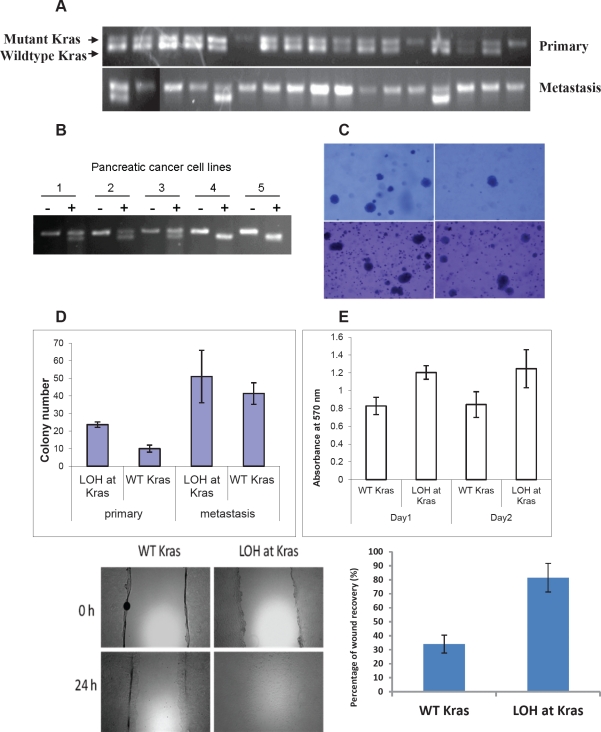
Loss of the wild-type allele of Kras is associated with invasiveness and metastasis of pancreatic tumors in *p16^flox/flox^*; *LSL-Kras^G12D^*; *Pdx1-Cre* mice (A) Representative samples showing loss of the wild-type allele of *Kras* (LOH at *Kras*) occurred more frequently in cancer cell lines derived from metastases than those derived from primary tumors of *p16*^*flox/flox*^; *LSL*-*Kras*^*G12D*^; *Pdx1-Cre* mice by PCR of genomic DNA, and confirmed by. (B) LOH at *Kras* was confirmed by RT-PCR and restriction fragment length polymorphism analyses [18]. PCR-amplified cDNA was untreated (-) or digested with HindIII (+). Representative pancreatic cancer cell lines 1 to 3 contained both wild-type (upper band) and mutant *Kras* alleles (lower band), whereas cell lines 4 and 5 lacked the wild-type *Kras* allele. (C, D) In colony formation assay with soft agar, primary pancreatic cancer cell lines with LOH at *Kras* formed significantly more colonies (23.7± 1.5) than those retained the *Kras* allele (10.0±2.0) (p<0.05). There was no significant difference between metastatic cells with or without LOH at *Kras* (51.0±14.9 vs. 41.3±6.1; P>0.05). (E) LOH at *Kras* also promoted cellular proliferation of the primary cancer cell lines in low serum environment at day 1(P<0.01) and day 2 (p<0.05) by MTT assay. (F, G) Increased migration was also correlated with LOH at *Kras* as measured by percentage of wound healing at 24 hours. The data represent the mean ± SD (n=3, p< 0.05).

*LSL-Kras*^*G12D*^ was conditionally activated in all of the pancreatic cancer cell lines (Fig. 5A), indicating that Kras activation is required for pancreatic tumorigenesis in these mice. Surprisingly, some of the cell lines possessed only the recombined *Kras* allele, but not the wild-type *Kras* allele (Fig. 5A). RT-PCR/RFLP analysis of RNA confirmed that the wild-type allele of *Kras* was lost (LOH at *Kras*) in some tumor cell lines but never the mutant *Kras*^*G12D*^ allele (Fig. 5B). Comparing the cell lines derived from the two paired primary tumors and metastases derived from the same mice, the frequency of LOH at *Kras* was greater among the cancer cell lines derived from each metastasis (8.3% and 33.3%) than those derived from the corresponding primary pancreatic tumor (0% and 17.6%, respectively) (p=0.16 and p<0.05, respectively) (Fig. 5A), suggesting *Kras* LOH might promote metastasis *in vivo*. We also microdissected liver metastases to confirm that LOH at *Kras* indeed occurred *in vivo*, and was not an artifact generated by tissue culture adaptation (Fig. S6).

To investigate if LOH at *Kras* has any phenotypic consequences, cancer cell lines derived from the primary tumor and metastasis of the same *p16*^*flox/flox*^; *LSL- Kras*^*G12D*^; *Pdx1-Cre* mouse were subjected to *in vitro* assays. In soft-agar colony formation assays, clonal cell lines of the primary tumor with LOH at *Kras* (23.7±1.5) formed more colonies than those without LOH at *Kras* (10.0±2.0) (p<0.05). The numbers of colonies formed by cell lines from the metastasis were generally greater than those of the primary tumor cells, but the difference between the metastatic cell lines with or without LOH at *Kras* (51.0±14.9 *vs*. 41.3±6.1 respectively) was not statistically significant (Fig. 5C, D). Under low serum condition, significant increased proliferation was observed in primary tumor cell lines with LOH at *Kras* than those without (Fig. 5E). Loss of the wild-type *Kras* allele also promoted motility among the primary cancer cell lines significantly (Fig 5F, G). Real-time PCR did not detect amplification of the mutant *Kras* allele in primary or metastatic cancer cell lines and confirmed the loss of the wild-type *Kras* allele (Table S3); indicating that the functional differences observed in these cancer cell lines were not caused by differential mutant *Kras* amplification, but rather by the loss of the wild-type *Kras* allele. We investigated whether the MAPK signaling pathway is responsible for the functional differences observed in primary and metastatic cancer cell lines with or without LOH at *Kras* and found no discernible difference of ERK1/2 phosphorylation in cancer cell lines with or without LOH of *Kras* by western blot analyses.

**Figure 6:**
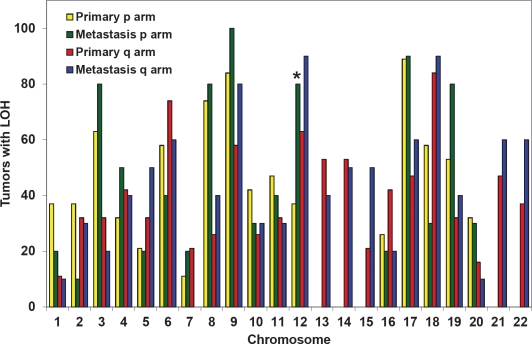
Increased LOH at KRAS (chromosome 12p) in human metastases compared with primary pancreatic cancers SNP analyses were performed on human cancer cell lines derived from pancreatic primary tumors (n=19) or metastases (n=10). High frequency allelic loss was exclusively observed at chromosome 12p (where *KRAS* is located) in metastatic cells, but not primary tumor cells (p<0.02).

Our *in vivo* and *in vitro* data suggested that LOH at *Kras* rendered primary pancreatic tumor cells more aggressive functional phenotypes that favored growth and metastasis in mice. To investigate if such phenomena also occurs in humans, non-biased whole-genome LOH profiles were generated from human cancer cell lines derived from primary pancreatic tumors (n=19) or metastases (n=10). There was no statistical difference in the LOH profiles of the primary tumors *vs.* metastases except at chromosome 12p, which contains the *KRAS* gene. LOH at chromosome 12p was observed in 37% of primary and 80% of metastatic cancer cell lines (p<0.02) (Fig. 6). LOH at *KRAS* was confirmed by genomic sequencing of the *KRAS* gene in the cancer cell lines with LOH at chromosome 12p (Fig. S7). The lack of significant difference in the allelic loss on other chromosomes strongly supports that the increased LOH at chromosome 12p is a selective and targeted event. These data corroborates the observations made in our mice, suggesting that LOH at *KRAS* is associated with metastasis and may promote metastasis.

## DISCUSSION

We reported the first mouse line with conditional knockout of *p16* that does not alter *p19* expression (Fig. 1, S6). This mouse strain is distinct from the previously reported *p16/p19* knockout strains [18, 21]. These mice are useful for investigating the functions of p16, without disrupting the p19-Mdm2-p53 pathway, in tissue-specific manners.

In combination with expression of *Kras*^*G12D*^, pancreas-specific disruption of *p16* in *LSL- Kras*^*G12D*^*; Pdx1-Cre* mice promoted progression of mPanIN to invasive pancreatic ductal adenocarcinoma and significantly shorten their median survival from >13 months to 4-6 months (Fig. 2) [25]. The *p16*^*−/−*^*; LSL- Kras*^*G12D*^*; Pdx1-Cre* mice and *p16*^*flox/flox*^*; LSL- Kras*^*G12D*^*; Pdx1-Cre* mice had median survival times of 4 and 6 months respectively—longer than the 2-month average survival time reported for *p16/p19*^*flox/flox*^*; LSL- Kras*^*G12D*^*; Pdx1-Cre* and *p16/p19*^*−/−*^*; LSL- Kras*^*G12D*^*; Pdx1-Cre* mice [18, 21]. The survival curves of *p16/p19*^*flox/flox*^*; LSL- Kras*^*G12D*^*; Pdx1-Cre* and *p16/p19*^*−/−*^*; LSL- Kras*^*G12D*^*; Pdx1-Cre* mice more closely resemble those of *p53*^*flox/flox*^*; LSL- Kras*^*G12D*^*; Pdx1-Cre* mice, perhaps due to the fact that *p19* and *p53* belong in the same pathway. Therefore it is important to differentiate the contributions of *p16* and *p19* to pancreatic tumorigenesis.

Similar mPanIN progression profile was observed in *p16*^*−/−*^*; LSL- Kras*^*G12D*^*; Pdx1-Cre* and *p16*^*flox/flox*^*; LSL- Kras*^*G12D*^*; Pdx1-Cre* mice as in *LSL-Kras*^*G12D*^*; PDX1-Cre* mice but at an accelerated pace (Fig. 4), indicating that biallelic inactivation of *p16* accelerated the development of PDA in synergy with *Kras*^*G12D*^ but did not alter the pathological progression of the pancreatic lesions- 20% of the pancreatic ducts in *p16*^*−/−*^*; LSL- Kras*^*G12D*^*; Pdx1-Cre* mice had mPanIN presentations around 4 weeks of age (Fig. 4), as opposed to >2 months in *LSL-Kras*^*G12D*^*; PDX1-Cre* mice [11]. The mPanIN lesions evolved and progressed to invasive cancer, and the most common histologic variant was comprised predominantly of malignant glands characterizing a well to moderately differentiated adenocarcinoma (Fig. 2, S3, Tables S1, S2). This is in contrast to the undifferentiated sarcomatoid histology previously reported in *p16*^*−/−*^*; LSL- Kras*^*G12D*^*; Pdx1-Cre* mice (n=3) [21]. The difference may simply be in the number of mice examined, but nevertheless, given that the majority of human pancreatic cancer is presented as well to moderately differentiated ductal adenocarcinoma, it's important that we have demonstrated here that biallelic inactivation of *p16* in conjunction with oncogenic *Kras* in mice mimic human pancreatic tumor progression from PanIN to PDA.

A striking feature of the *p16*^*−/−*^*; LSL- Kras*^*G12D*^*; Pdx1-Cre* and *p16*^*flox/flox*^*; LSL- Kras*^*G12D*^*; Pdx1-Cre* mice was the high penetrance of metastases. Metastases were observed in 100% of the non-thriving *p16*^*−/−*^*; LSL- Kras*^*G12D*^*; Pdx1-Cre* mice (n=14). It has been reported previously that only 63% of *p53*^*R172H/+*^*; LSL-KrasS*^*G12D*^*; PDX1-Cre* mice developed metastasis [25, 26], 33% was detected in *p53*^*lox/+*^*; LSL-Kras*^*G12D*^*; PDX1-Cre* mice, and none was reported in *p53*^*lox/lox*^*; LSL-Kras*^*G12D*^*; PDX1-Cre* mice [21, 26]. It appears that inactivation of *p16* in the presence of activated *Kras* is associated with increased metastasis in comparison to *p53* inactivation; however, the mechanism behind this difference is not known presently. This difference in metastasis frequency cannot be explained by mere difference in medium survival- the reported medium survival of *p53*^*R172H/+*^*; LSL-KrasS*^*G12D*^*; PDX1-Cre* mice (5 months) [25] is longer than that of *p16*^*−/−*^*; LSL- Kras*^*G12D*^*; Pdx1-Cre* mice. The frequent and consistent metastases observed in the *p16*^*−/−*^*; LSL- Kras*^*G12D*^*; Pdx1-Cre* and *p16*^*flox/flox*^*; LSL- Kras*^*G12D*^*; Pdx1-Cre* mice make them ideal models for studying advanced human PDA and metastasis.

Numerous studies in mouse models have shown that *Kras*^*G12D*^ is sufficient to initiate development of PanINs and their progression to invasive cancer [6, 10, 11]. Here we show that that in addition to its role in tumor initiation, *Kras*^*G12D*^ may promote tumor progression and metastasis. The loss of the wild-type allele of *Kras* during the progression from primary carcinomas to metastases suggests that its loss might confer growth advantage in the presence of oncogenic e *Kras*^*G12D*^ allele. LOH at *Kras* did not appear to be a random event, because only the wild-type *Kras* allele was selectively lost, which resulted in discernible functional phenotypes (Fig. 5). LOH at *Kras* was detectable in liver metastases and not a mere result of tissue culture adaptation (Fig. S6). LOH at *Kras* was not an artifact created by genetic engineering or unique to mice, because an increased frequency of LOH at *KRAS* was observed in human metastatic cell lines (80%) compared with primary cancer cell lines (37%) (Fig. 6). *KRAS* is located on chromosome 12p—the only chromosomal region in which a statistically significant difference was observed between human primary and metastatic cancer cells. The absence of increased LOH at other chromosomal arm strongly supports that the LOH at *KRA*S is not simply a manifestation of increased genomic stability during progression. Previous studies proposed that a wild-type allele of *Kras* allele can serve as a tumor-suppressor [7, 12, 27-30]. Our data demonstrate that sporadic loss of the wild-type *Kras* allele occurred in pancreatic tumors and metastases in both mice and humans. Our data is the first to provide *in vivo* and *in vitro* evidence that LOH at wild-type *Kras* is associated with pancreatic tumor metastasis.

The functional impacts of oncogenic Kras in tumor initiation are well known, but the potential role of wild-type Kras in this process remains elusive. It is possible that the loss of wild-type Kras merely serves to remove a competitor for limited resources, such as GTP. However, many previous studies have suggested that the dynamics between the wild-type and mutant Kras proteins is complex and the expression level of the mutant *Kras* allele and/or the ratio of the wild-type to mutant Kras proteins are critical to tumorigenesis [6-10, 12, 13]. Identification of differential downstream effector proteins for wild-type and oncogenic KRAS in the future will help elucidating if the wild-type *KRAS* allele possesses any tumor-suppressive function. However, regardless what the underlying molecular mechanism is, our current study already has a strong implication on the development KRAS target therapy. The current thinking on KRAS target therapy does not differentiate between inhibiting wild-type or oncogenic KRAS. Our data strongly recommends that future KRAS target therapies should specifically inhibit only the oncogenic KRAS, not the wild-type KRAS, because the non-discriminatory inhibition of wild-type KRAS may promote metastasis and produce unintended negative impacts on patient care.

We show that biallelic inactivation of the *p16* and *Kras*^*G12D*^ act synergistically to promote pancreatic tumorigenesis and metastasis. The *p16*^*flox/flox*^*; LSL- Kras*^*G12D*^*; Pdx1-Cre* mice develop PanIN, invasive tumors, and metastases in a manner that recapitulates human PDA development and progression. The relative short median survival and high incidence of metastasis in these mice make them an attractive model for testing novel therapeutics and investigating metastasis—2 areas of research that are a challenge to explore in humans.

## MATERIALS AND METHODS

### Genetically engineered mouse models and mouse strains

To generate a conditional p16 knockout mouse line (*p16*^*flox/flox*^), exon1α of *p16* was flanked by loxP sites, leaving *p19* expression intact (Fig. S1 and 1). The details on the generation of the *p16*^*flox/flox*^ mouse line are described in the Supplementary Methods. The resultant *p16*^*flox/flox*^ mice were of mixed 129/SvJ and C57BL/6 backgrounds. The *p16*^*−/−*^ [22] and *LSL- Kras*^*G12D*^ [11] mice were obtained through the Mouse Models of Human Cancers Consortium Repository. *Pdx1-Cre* mice were previously described [11]. All studies were conducted in compliance with the IACUC guidelines of Columbia University.

### Histology and immunohistochemistry

Tissues were fixed in 10% formalin overnight and embedded in paraffin. The detailed protocol and antibodies for immunohistochemistry are described in the Supplementary Methods.

### Establishment and cultivation of primary pancreatic adenocarcinoma cell lines

Freshly isolated murine tumor specimens were minced with sterile razor blades, digested with 0.5% trypsin for 15 minutes at 37°C, resuspended in DMEM and 15% fetal bovine serum (FBS), and seeded in 10 cm dishes. All genetic analyses were performed on cells cultivated for less than 7 passages. Each primary cancer cell culture was then sorted by flow cytometry and seeded at a single cell per well, into 96-well tissue-culture plates, to establish clonal cell lines. About 18 to 50 clonal cell lines were obtained from each original primary cancer cell line.

### Immunoblot analysis

Tissues or cell pellets were lysed in 20 mM Tris (pH 7.5), 150 mM NaCl, 1mM EDTA, 1mM EGTA, and 1% Triton X-100 in the presence of a protease inhibitor cocktail (Roche, Indianapolis, IN) or and a phosphatase inhibitor cocktail (kits I and II, Calbiochem). Immunoblotting was performed as described previously [31, 32] and as in the Supplementary Methods.

### Colony formation, MTT, and migration assays

These assays were performed with standard protocols and briefly described in the Supplementary Methods. All experiments were performed in triplicate and repeated at least 3 times.

### SNP analyses of human cancer cell lines

Twenty-nine human cancer cell lines were used, and segregated into those arising from primary carcinomas (BxPc3, MiaPaCa-2, Panc-1, PK9, PL-1, PL-5, PL-9, PL-11, PL-13, PL-19, PL20, PL21, PL22, PL23, PL24, XPA1, XPA2, XPA3, and A13A/B) or from pancreatic cancer metastases (AsPc1, CAPAN1, CFPAC1, Hs766T, PK-8, Su86.86, A6L, A2.1, A13D, and A10.7) [33, 34]. The allelotypes of 24 of these cell lines were described previously [33], whereas those of cell lines A13A/B, A6L, A2.1, A13D and A10.7 were generated for this study. The genotypes of 115,353 SNPs were analyzed in all cell lines using Affymetrix oligonucleotide arrays hybridized to reduced complexity genomic DNA as previously described [35]. Genotypes were determined by the GeneChip® DNA Analysis Software Tool (GDAST, v3.0) using a 0.05 quality score setting genotypes were plotted with respect to their genomic position listed in the May 2004 assembly of the human genome and briefly described in the Supplementary Method.

### Statistical analysis

Results were presented as the mean ± SD. the Student *t* test was used to compare data between groups and the χ2 method was used to compare the frequency of allelic loss among cell lines. P values less than 0.05 were considered to be statistically significant.

## Supplementary Figures, Meathods and Tables







## References

[1] Jemal A, Siegel R, Ward E, Hao Y, Xu J, Thun MJ (2009). Cancer statistics, 2009. CA Cancer J Clin.

[2] Kern S, Hruban R, Hollingsworth MA, Brand R, Adrian TE, Jaffee E, Tempero MA (2001). A white paper: the product of a pancreas cancer think tank. Cancer Res.

[3] Almoguera C, Shibata D, Forrester K, Martin J, Arnheim N, Perucho M (1988). Most human carcinomas of the exocrine pancreas contain mutant c-K-ras genes. Cell.

[4] Smit VT, Boot AJ, Smits AM, Fleuren GJ, Cornelisse CJ, Bos JL (1988). KRAS codon 12 mutations occur very frequently in pancreatic adenocarcinomas. Nucleic Acids Res.

[5] Hruban RH, Wilentz RE, Kern SE (2000). Genetic progression in the pancreatic ducts. Am J Pathol.

[6] Guerra C, Mijimolle N, Dhawahir A, Dubus P, Barradas M, Serrano M, Campuzano V, Barbacid M (2003). Tumor induction by an endogenous K-ras oncogene is highly dependent on cellular context. Cancer Cell.

[7] Zhang Z, Wang Y, Vikis HG, Johnson L, Liu G, Li J, Anderson MW, Sills RC, Hong HL, Devereux TR (2001). Wildtype Kras2 can inhibit lung carcinogenesis in mice. Nat Genet.

[8] Brembeck FH, Schreiber FS, Deramaudt TB, Craig L, Rhoades B, Swain G, Grippo P, Stoffers DA, Silberg DG, Rustgi AK (2003). The mutant K-ras oncogene causes pancreatic periductal lymphocytic infiltration and gastric mucous neck cell hyperplasia in transgenic mice. Cancer Res.

[9] Grippo PJ, Nowlin PS, Demeure MJ, Longnecker DS, Sandgren EP (2003). Preinvasive pancreatic neoplasia of ductal phenotype induced by acinar cell targeting of mutant kras in transgenic mice. Cancer Res.

[10] Tuveson DA, Shaw AT, Willis NA, Silver DP, Jackson EL, Chang S, Mercer KL, Grochow R, Hock H, Crowley D (2004). Endogenous oncogenic K-ras(G12D) stimulates proliferation and widespread neoplastic and developmental defects. Cancer Cell.

[11] Hingorani SR, Petricoin EF, Maitra A, Rajapakse V, King C, Jacobetz MA, Ross S, Conrads TP, Veenstra TD, Hitt BA (2003). Preinvasive and invasive ductal pancreatic cancer and its early detection in the mouse. Cancer Cell.

[12] Li J, Zhang Z, Dai Z, Plass C, Morrison C, Wang Y, Wiest JS, Anderson MW, You M (2003). LOH of chromosome 12p correlates with Kras2 mutation in non-small cell lung cancer. Oncogene.

[13] To MD, Wong CE, Karnezis AN, Del Rosario R, Di Lauro R, Balmain A (2008). Kras regulatory elements and exon 4A determine mutation specificity in lung cancer. Nat Genet.

[14] Jones S, Zhang X, Parsons DW, Lin JC, Leary RJ, Angenendt P, Mankoo P, Carter H, Kamiyama H, Jimeno A (2008). Core signaling pathways in human pancreatic cancers revealed by global genomic analyses. Science.

[15] Schutte M, Hruban RH, Geradts J, Maynard R, Hilgers W, Rabindran SK, Moskaluk CA, Hahn SA, Schwarte-Waldhoff I, Schmiegel W (1997). Abrogation of the Rb/p16 tumor-suppressive pathway in virtually all pancreatic carcinomas. Cancer Res.

[16] Rozenblum E, Schutte M, Goggins M, Hahn SA, Lu J, Panzer S, Zahurak M, Goodman SN, Hruban RH, Yeo CJ (1997). Tumor-suppressive pathways in pancreatic carcinoma. Cancer Res.

[17] Serrano M, Lee H, Chin L, Cordon-Cardo C, Beach D, DePinho RA (1996). Role of the INK4a locus in tumor suppression and cell mortality. Cell.

[18] Aguirre AJ, Bardeesy N, Sinha M, Lopez L, Tuveson DA, Horner J, Redston MS, DePinho RA (2003). Activated Kras and Ink4a/Arf deficiency cooperate to produce metastatic pancreatic ductal adenocarcinoma. Genes Dev.

[19] Lowe SW, Sherr CJ (2003). Tumor suppression by Ink4a-Arf: progress and puzzles. Curr Opin Genet Dev.

[20] Pomerantz J, Schreiber-Agus N, Liegeois NJ, Silverman A, Alland L, Chin L, Potes J, Chen K, Orlow I, Lee HW (1998). The Ink4a tumor suppressor gene product, p19Arf, interacts with MDM2 and neutralizes MDM2′s inhibition of p53. Cell.

[21] Bardeesy N, Aguirre AJ, Chu GC, Cheng KH, Lopez LV, Hezel AF, Feng B, Brennan C, Weissleder R, Mahmood U (2006). Both p16(Ink4a) and the p19(Arf)-p53 pathway constrain progression of pancreatic adenocarcinoma in the mouse. Proc Natl Acad Sci U S A.

[22] Sharpless NE, Bardeesy N, Lee KH, Carrasco D, Castrillon DH, Aguirre AJ, Wu EA, Horner JW, DePinho RA (2001). Loss of p16Ink4a with retention of p19Arf predisposes mice to tumorigenesis. Nature.

[23] Maitra A, Adsay NV, Argani P, Iacobuzio-Donahue C, De Marzo A, Cameron JL, Yeo CJ, Hruban RH (2003). Multicomponent analysis of the pancreatic adenocarcinoma progression model using a pancreatic intraepithelial neoplasia tissue microarray. Modern Pathology.

[24] Maitra A, Fukushima N, Takaori K, Hruban RH (2005). Precursors to invasive pancreatic cancer. Adv Anat Pathol.

[25] Hingorani SR, Wang L, Multani AS, Combs C, Deramaudt TB, Hruban RH, Rustgi AK, Chang S, Tuveson DA (2005). Trp53R172H and KrasG12D cooperate to promote chromosomal instability and widely metastatic pancreatic ductal adenocarcinoma in mice [see comment]. Cancer Cell.

[26] Morton JP, Timpson P, Karim SA, Ridgway RA, Athineos D, Doyle B, Jamieson NB, Oien KA, Lowy AM, Brunton VG Mutant p53 drives metastasis and overcomes growth arrest/senescence in pancreatic cancer. Proc Natl Acad Sci U S A.

[27] Hegi ME, Devereux TR, Dietrich WF, Cochran CJ, Lander ES, Foley JF, Maronpot RR, Anderson MW, Wiseman RW (1994). Allelotype analysis of mouse lung carcinomas reveals frequent allelic losses on chromosome 4 and an association between allelic imbalances on chromosome 6 and K-ras activation. Cancer Res.

[28] Bremner R, Balmain A (1990). Genetic changes in skin tumor progression: correlation between presence of a mutant ras gene and loss of heterozygosity on mouse chromosome 7. Cell.

[29] Li H, Cao HF, Wan J, Li Y, Zhu ML, Zhao P (2007). Growth inhibitory effect of wild-type Kras2 gene on a colonic adenocarcinoma cell line. World J Gastroenterol.

[30] Fleming JB, Shen GL, Holloway SE, Davis M, Brekken RA (2005). Molecular consequences of silencing mutant K-ras in pancreatic cancer cells: justification for K-ras-directed therapy. Molecular Cancer Research: MCR.

[31] Qiu W, Schonleben F, Thaker HM, Goggins M, Su GH (2006). A novel mutation of STK11/LKB1 gene leads to the loss of cell growth inhibition in head and neck squamous cell carcinoma. Oncogene.

[32] Qiu W, Schonleben F, Li X, Su GH (2007). Disruption of transforming growth factor beta-Smad signaling pathway in head and neck squamous cell carcinoma as evidenced by mutations of SMAD2 and SMAD4. Cancer Lett.

[33] Calhoun ES, Hucl T, Gallmeier E, West KM, Arking DE, Maitra A, Iacobuzio-Donahue CA, Chakravarti A, Hruban RH, Kern SE (2006). Identifying allelic loss and homozygous deletions in pancreatic cancer without matched normals using high-density single-nucleotide polymorphism arrays. Cancer Research.

[34] Fu B, Guo M, Wang S, Campagna D, Luo M, Herman JG, Iacobuzio-Donahue CA (2007). Evaluation of GATA-4 and GATA-5 methylation profiles in human pancreatic cancers indicate promoter methylation patterns distinct from other human tumor types. Cancer Biol Ther.

[35] Matsuzaki H, Dong S, Loi H, Di X, Liu G, Hubbell E, Law J, Berntsen T, Chadha M, Hui H (2004). Genotyping over 100,000 SNPs on a pair of oligonucleotide arrays. Nat Methods.

