# Supplementary material for: Disruption of p16 and Activation of Kras in Pancreas Increase Ductal Adenocarcinoma Formation and Metastasis in vivo
Source: Oncotarget. 2011 Nov 23;2(11):862–73. doi: 10.18632/oncotarget.357 (PMC3259996; doi:10.18632/oncotarget.357)
Supplement: Supplementary file 1 [file oncotarget-02-862-s001.pdf]

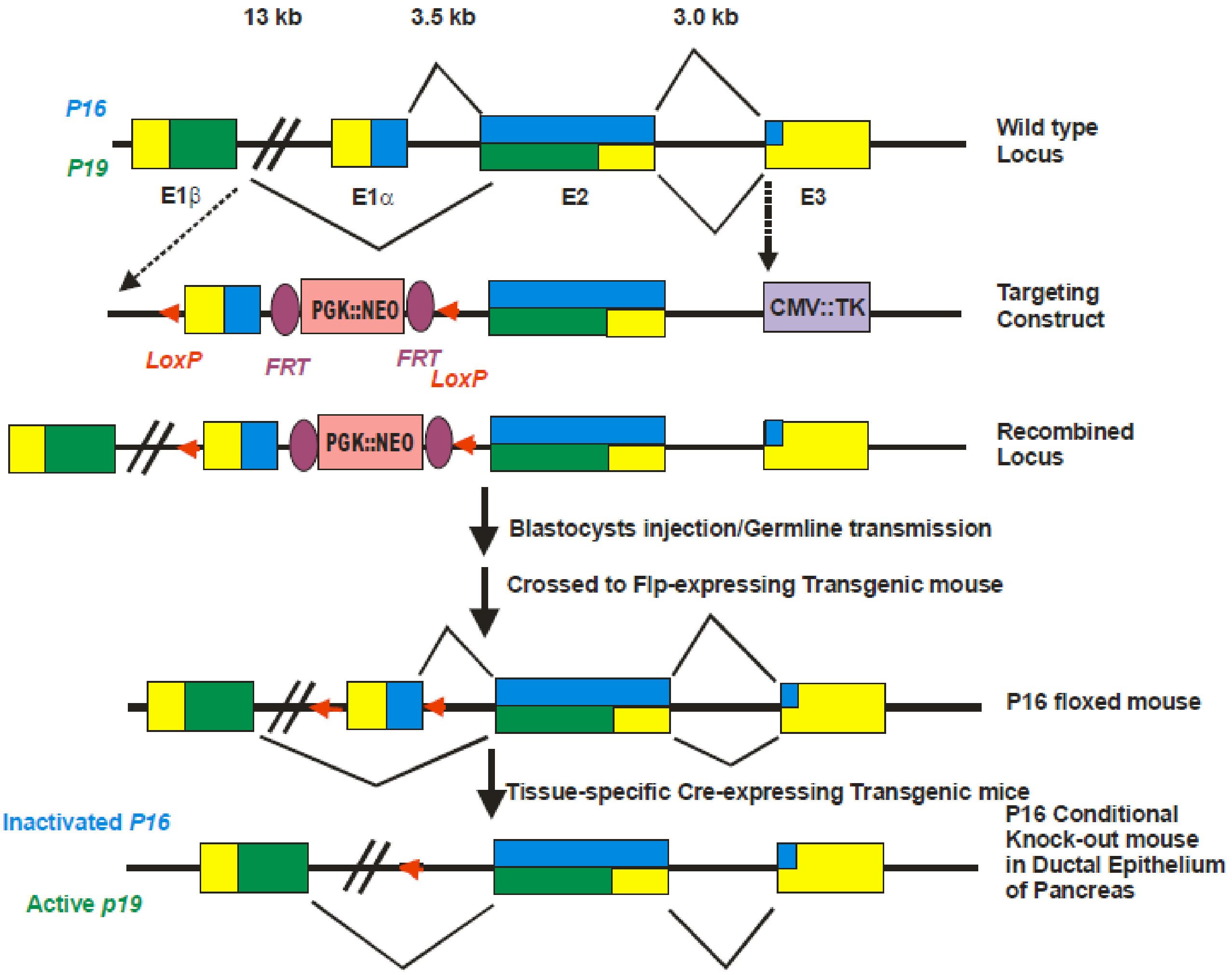

Figure S1. Conditional inactivation of the *p16* gene with retaining *p19* expression. Exon 1α of the *p16* wild-type locus is not shared with p19. The targeting construct contained two *loxP* sites (arrows), two *Frt* sequences (ovals), a *Neo* cassette for positive selection, and a *thymidine kinase* cassette for negative selection. Exon 1α of the *p16* gene was flanked by two *loxP* sites. The homologous recombination of the targeted mouse *p16* allele in ES cells is depicted here. The *Neo* cassette was subsequently removed *in vivo* by breeding with a *CMV-Flp* transgenic mouse. The conditional deletion of p16 exon1α occurred somatically when bred to tissue-specific *Cre* transgenic mice.

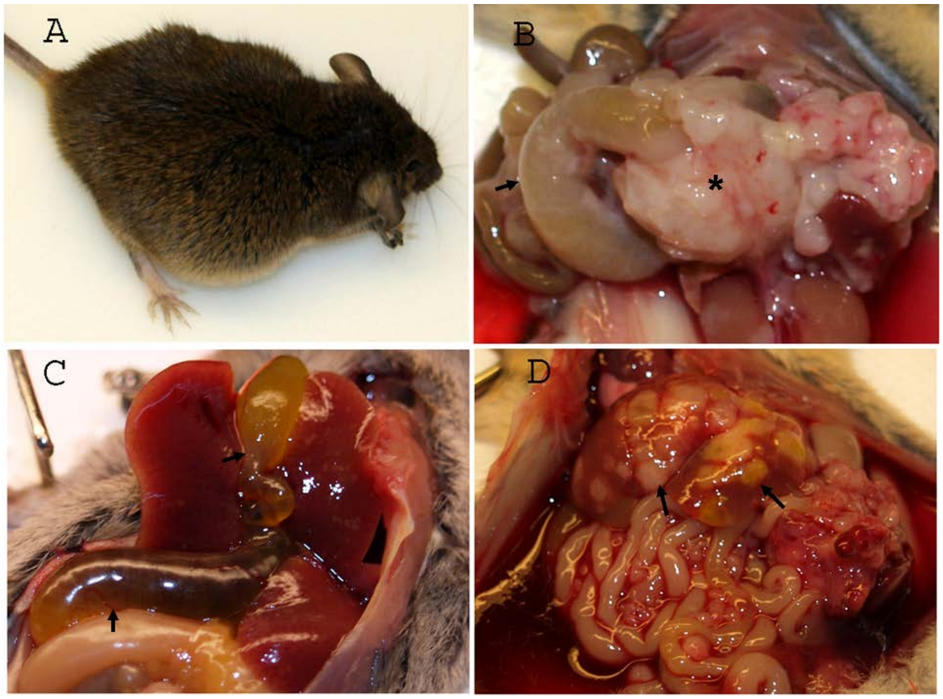

Figure S2. Representative pancreatic cancers of mutant *p16*$^{-/-}$; *LSL- Kras*^{G12D}; *Pdx1-Cre* mice that mimic human PDA. (A) Three-month-old mutant mouse with abdominal distension due to malignant ascites. (B) Three-month-old mutant mouse with advanced PDA involving the entire pancreas (asterisk), which invaded surrounding tissues with resultant small bowel distension due to direct invasion and compression of the proximal duodenum. (C) Three-month-old mutant mouse with marked dilation of the common bile duct and gallbladder distension (arrow) due to a pancreatic tumor in the head (unlabeled). (D) A four-month-old mouse was shown here with multiple metastases (arrow) distributed throughout the liver.

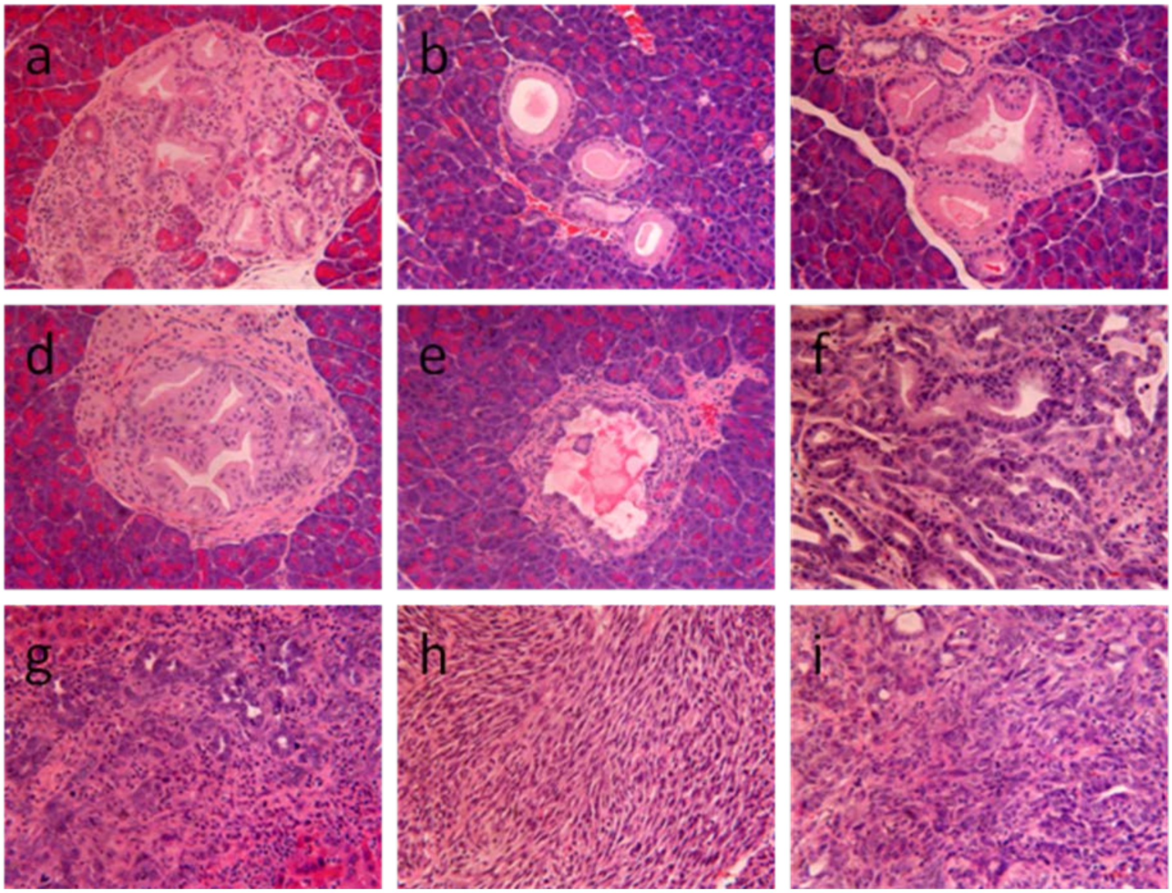

Figure S3. Histological analyses of pancreatic cancers in *p16^flox/flox^*; *LSL- Kras^G12D^*; *Pdx1-Cre* mice. The tumor progression pattern observed in *p16^flox/flox^*; *LSL- Kras^G12D^*; *Pdx1-Cre* mice is similar to those seen in *p16^-/-^*; *LSL- Kras^G12D^*; *Pdx1-Cre* mice. Early precancerous lesions, (a) acinar to ductal metaplasia, (b) mPanIN 1A, (c) mPanIN 1B, (d) mPanIN 2, and (e) mPanIN 3, were all detected. The majority of invasive cancers are presented as ductal adenocarcinoma, while sacarcomatoid and anaplastic adecarcinoma can also be seen. Here shown are (f) moderately differentiated ductal adenocarcinoma in a 3 month old mouse, (g) liver metatstasis in the same mouse, (h) sarcomatoid adenocarcinoam in a 4 month old mouse, and (i) anaplastic adenocarcinoma in a 3 month old mouse.

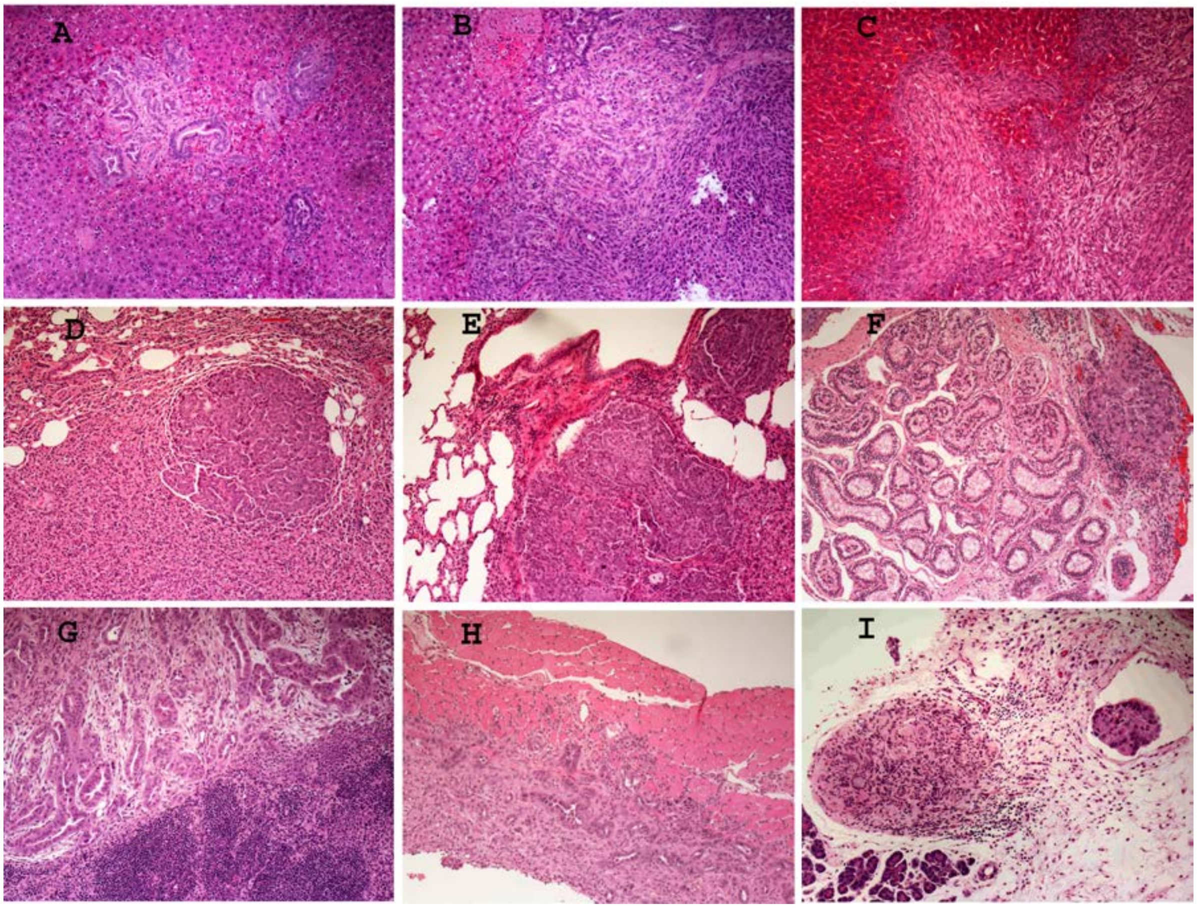

Figure S4. Histological analyses of metastases of pancreatic cancers in *p16*$^{-/-}$; *LSL- Kras*^{G12D}; *Pdx1-Cre* mice. (A) The malignant glandular component of PDA was frequently observed in liver metastasis. Anaplastic (B) and sarcomatoid (C) components were also seen in liver metastases. Pancreatic cancer metastases to the lung (D, E), testis (F), spleen (G), stomach muscle (H), and lymphatic vessels (I) were also observed. A lung ductal adenoma, possibly a second primary tumor, was observed alongside of pancreatic metastasis in the lung (D).

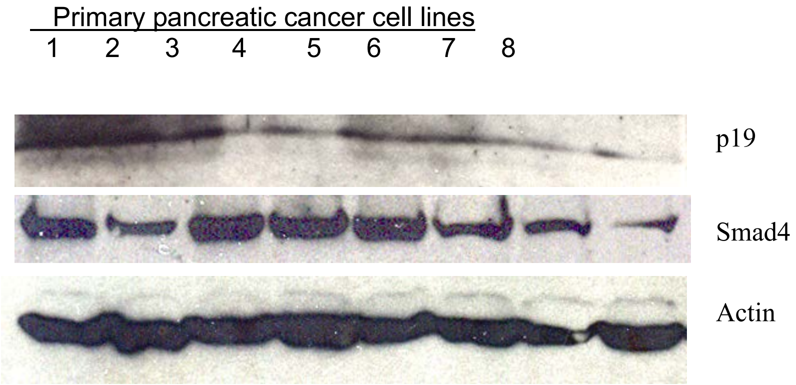

Figure S5. Smad4 and p19 protein expressions remained intact in pancreatic cancer cell lines derived from the tumors of *p16*$^{flox/flox}$; *LSL-Kras*^{G12D}; *Pdx1-Cre* mice. Total protein lysates were blotted with antibody against p19 or Smad4 using western blot analysis. Smad4 and p19 proteins were detected in all cancer cell lines examined.

A.

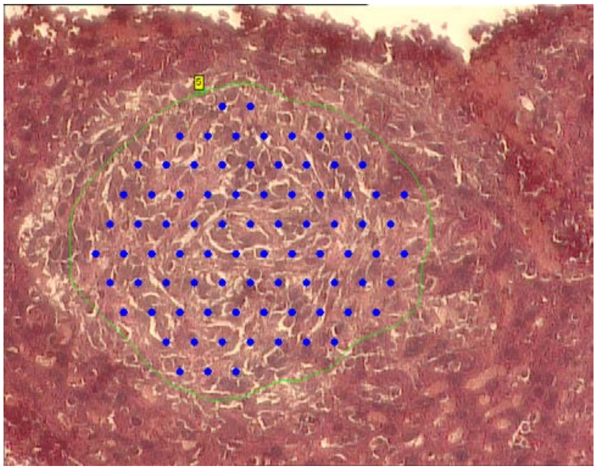

B

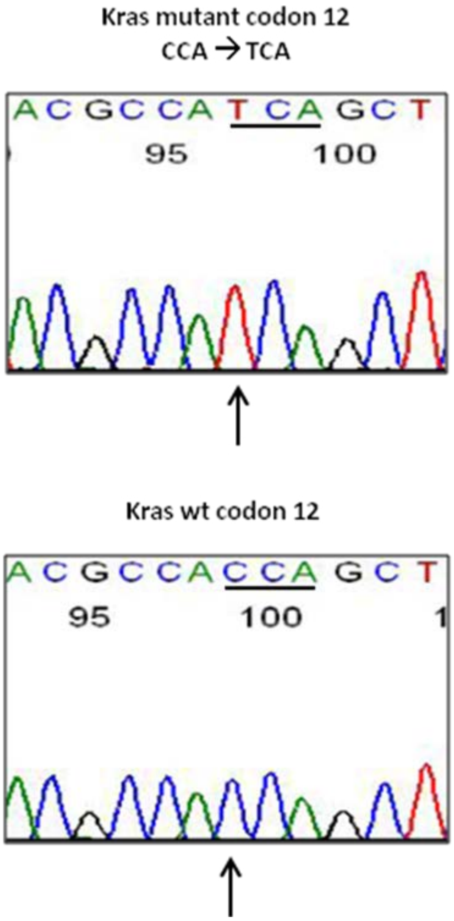

Figure S6. LOH at Kras was detected in the liver metastases of *p16$^{flox/flox}$; LSL-Kras^{G12D}; Pdx1-Cre* mice. Multiple loci of liver metastases were microdissected for DNA analyses. LOH at Kras was determined by PCR and sequencing to reveal the loss of the wild-type Kras allele. A) A representative laser microdissected metastatic locus. B) The corresponding sequencing result of the microdissected locus and a normal tissue control.

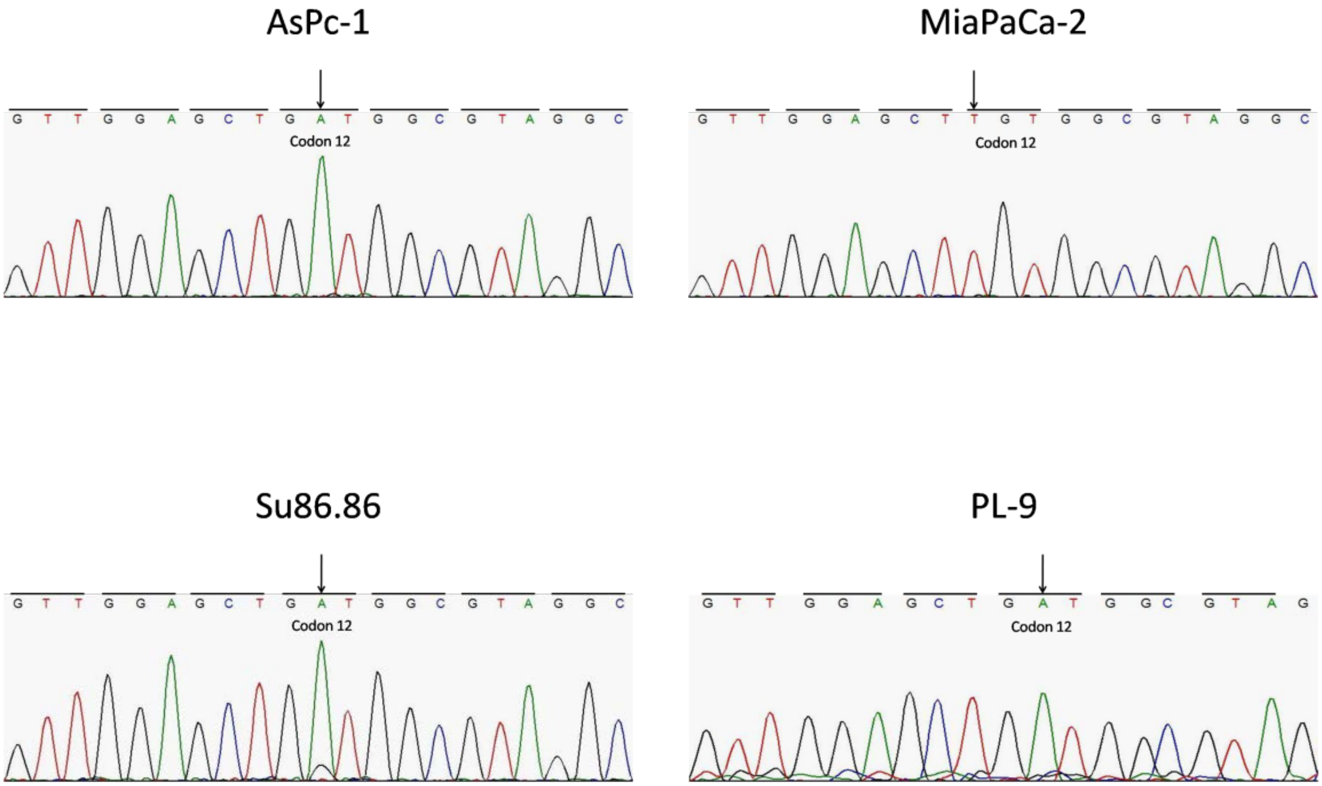

Figure S7. Genomic sequencing of the *KRAS* gene confirmed LOH at *KRAS*. Representatives of genomic sequencing of *KRAS* are shown here displaying the mutant *KRAS* allele at codon 12 and the absence of the wild-type *KRAS* allele. The four cell lines shown here all have LOH at chromosome 12p by the SNP analyses. The sequencing data confirms that LOH at chromosome 12p included the *KRAS* gene locus and resulted in the loss of the wild-type *KRAS* allele.
