# Supplementary material for: Disruption of p16 and Activation of Kras in Pancreas Increase Ductal Adenocarcinoma Formation and Metastasis in vivo
Source: Oncotarget. 2011 Nov 23;2(11):862–73. doi: 10.18632/oncotarget.357 (PMC3259996; doi:10.18632/oncotarget.357)
Supplement: Supplementary file 2 [file oncotarget-02-862-s002.pdf]

Disruption of *p16* and Activation of *Kras* in Pancreas Increases Ductal Adenocarcinoma Formation and Metastasis *in vivo*

SUPPLEMENTARY METHODS

Construction of the conditional p16 targeting vector without affecting p19 expression. A 129/Sv mouse genomic library (Stratagene; Lambda FIXII) was screened with a mouse p16 cDNA probe which contain all *p16* encoding area. A lambda clone, which contains an around 14 kb genomic fragment including all *p16* exons, was used to construct the conditional p16 targeting vector. A 1927-bp of genome between EcoRI and XbaI containing *p16* exon1α was linkered with HindIII and cloned in HindIII site as flanked by two Cre-loxP sites of vector pNeo-FRT-loxP (a gift of Drs. Kogo Takamiya and Richard L. Huganir at The Johns Hopkins University). Two additional fragments were also derived from the phage. *p16* short arm from the EcoR1 to 5' site of gene derived from phage is around 1400bp and long arm obtained between Xba1-NotI is 6867bp. Short and long arm fragments were inserted into pNeo-FRT-LoxP vector subsequently. The construct was designed so that Flp-mediated recombination would remove the Neo-cassette from the targeting allele *in vivo*, and Cre-mediated recombination would lead to the deletion of a 1927-bp genomic sequence (EcoRI-XbaI fragment) of *p16* containing exon1α. This recombination would inactivate *p16* expression in a tissue-specific manner without disturbing *p19* transcription or translation (Fig. S1).

Electroporation and selection of ES cell clones. Mouse ES cells were transfected with a linearized targeting vector by electroporation and selected in G418 and gancyclovir containing medium. The ES cells were derived from 129 mice which have agouti coat colors. Homologous recombination was examined in G418-resistant ES clones by PCR, and positive clones were confirmed by Southern blot hybridization using a 3'-*p16* fragment external to the construct as the probe (data not shown). Positive recombinant clones were subsequently utilized for blastocyt microinjection.

Generation of chimeric mice and germline transmission of the *p16* floxed allele. ES clones that had undergone homologous recombination were microinjected into blastocyts derived from C57BL/6J mice. The blastocyst injection was performed by the Transgenic Core Facility at The Johns Hopkins University School of Medicine. The resulting chimeric males were backcrossed to C57BL/6J females, and F1 agouti offspring were analyzed for the presence of the conditional *p16* targeting allele. The Neo-cassette was subsequently removed from the germline-transmitted mice carrying the conditional *p16* targeting allele by mating with CMV-Flp transgenic mice (A gift of Dr. Thomas Ludwig at Columbia University Medical Center). The primers for PCR detection of the *FLP* gene are Flp-F: CTT CAA GCA ACA AGA AGG AA; Flp-R: TGC TTC CTT CAG CAC TAC CC. The primers used for the identification of the Neo-cassette are Neo-F: CTTCGTATAGCATACATTATACGAAG; and Neo-R: CGTGGATGTGGAATGTGTG. The removal of the Neo-cassette was further confirmed by PCR and DNA sequencing using primers P1: AGCAGCTTCTAATCCCAGCA and P2: CCACTCCTGGAACTCAGCAT (data not shown). The heterozygous floxed *p16* mice without Neo-cassette were interbred to generate homozygous p16-floxed mice.

DNA and RNA isolations from mouse tissues and genotypic analyses. DNA and RNA isolations from pancreatic tumor cell lines and primary tumors were performed by using Qiagen DNeasy tissue kit (Valencia, CA) and Invitrogen Trizol kit Carlsbad, CA), respectively.

Genotyping of the *p16*-floxed mice by PCR strategies through detecting of the 5' or 3' loxP loci were carried out with primers P1 and P2 or P3 (AGGAGTCCTGGCCCTAGAAA) and P4 (CCAAAGGCAAACTTCTCAGC) (Fig. 1). Mice homozygous for the floxed allele of *p16* were displayed as a single top band; two bands represented heterozygous *p16*-floxed mice; a single bottom band represented wild-type *p16* mice on gel electrophoresis (Fig. 1A, B). The detection of Cre-mediated recombination of the conditional *p16* allele was performed by PCR using primers P1 and P4 (Fig. 1A, C). Cre-mediated recombinations were further confirmed by DNA sequencing of randomly selected PCR-positive samples. Genotyping of the *Pdx1-Cre* mouse line was analyzed by PCR using primers Pdx1-cre-F: AGATGTTCGCGATTATCTTC; and Pdx1-cre-R: AGCTACACCAGAGACGG.

RT-PCR primers were designed to amplify the entire *p53* coding regions as a series of overlapping about 600bp DNA fragments. The sequences of these primer pairs were as follows: p53-mF1: GCTTCTCCGAAGACTGGATG; p53-mR1: CTCGGGTGGCTCATAAGGTA; p53-mF2: CGGGTGGAAGGAAATTTGTA; p53-mR2: TGCAGAGGCAGTCAGTCTGA.

PCR and RT-PCR/RFLP analyses employed to distinguish the wild-type *Kras* and activated $Kras^{G12D}$ mutant alleles and transcripts were modified from the primers described previously (12, 18). For RT-PCR/RFLP, cDNA samples are supposed to amplify a 243-bp product from both the wild-type and mutant transcripts. The $Kras^{G12D}$ allele but not the wild-type allele contains a HindIII restriction site engineered in exon1. Thus, digestion of the 243-bp PR-PCR product with HindIII yields 213-bp and 30-bp bands from the mutant product only.

Histology and immunohistochemistry. Tissues were fixed in 10% formalin overnight and embedded in paraffin. For immunohistochemistry, slides were deparaffinized in xylene and rehydrated sequentially in ethanol with rocking platform. Steaming in 0.01M citrate buffer (pH 6.0) for 30 minutes was used for antigen retrieval. Slides were quenched in peroxidase blocking reagent (Dako, Carpinteria, CA) for 5 minutes to block endogenous peroxidase activity. Then, the slides were incubated for one hour at room temperature with primary antibodies diluted in antibody diluent buffer (Dako). The Dako LSAB-System-HRP kit was employed for signal amplification. Finally slides were counterstained with hematoxylin, dehydrated sequentially in ethanol, cleared with xylenes, and mounted with Cytoseal 60. The antibodies and dilutions were: p53, 1:50 (CM5, Novocastra/Leica Microsystems, Bannockburn, IL); CK19, 1:20 (TROMA3, Developmental Studies Hybridoma Bank at the University of Iowa, Iowa City, Iowa); Insulin,1:1000 (A0564, Dako); Glucagon, 1:1000 (AB932, Millipore, Billerica, MA); p16, 1:50 (F-12, Santa Cruz Biotechnology, Santa Cruz, CA); Amylase, 1:1000 (Calbiochem/EMD Chemicals, Gibbstown, NJ); α-SMA, 1:100 (Lab Vision/Thermo Scientific, Fremont, CA); Cox2, 1:100 (cloneSP21, Thermo Scientific); Pdx-1, 1:100 (Millipore); Ki-67, 1:100 (Dako); p19, 1:50 (Millipore); Her2, 1:25 (Cell Signaling Technology, Danvers, MA); Smad4 (06-693, Millipore).

Western blot analysis. Tissues or cell pellets were lysed in 20 mM Tris (pH 7.5), 150 mM NaCl, 1mM EDTA, 1mM EGTA, and 1% Triton X-100 in the presence of a protease inhibitor cocktail (Roche, Indianapolis, IN) or and a phosphatase inhibitor cocktail (kits I and II, Calbiochem). Twenty five micrograms of lysate was resolved on 8%-12% Bis-Tris PAGE gels (Invitrogen) and transferred to nitrocellulose membranes. Membranes were blotted with p16 (M-156, Santa Cruz Biotechnology), Smad4 (B-8, Santa Cruz Biotechnology), $p19^{Arf}$ (Ab-80, Abcam, Cambridge, MA). For measurement of activated Ras level, the Ras activation assay kit (Upstate/Millipore) was used according to the manufacturer's instructions.

Colony formation, MTT, and migration assays. For the colony formation assay, $1x10^4$ cancer cells of either primary or metastatic mouse pancreatic cancer cell lines, with or without the wild-type *Kras* allele, were plated in 6-well plates with 0.3% agar. The cells were cultured for 2–3 weeks for colony formation. The colonies were stained with crystal violet and counted under a dissecting microscope. For the low serum proliferation assay, primary mouse pancreatic cancer cell lines, with or without the wild-type *Kras* allele, were cultured in 15% FBS/DMEM media and seeded at $5x10^3$ cells per well into 96-well culture plates in 1% FBS/DMEM. Cell proliferation was measured at 24 hours and 48 hours after switching to 1% FBS/DMEM media using the MTT assay kit (Roche). For migration assay, confluent monolayers of primary mouse pancreatic cancer cell lines, with or without the wild-type *Kras* allele, were scratched with a sterile pipette 10 tip. The plates were then washed and incubated at 37°C in 10%FBS/DMEM medium for 24 h. The rate of migration was calculated by measuring the distance moved toward the center of the wound after 24 hours.

Gene amplification analysis and real-time PCR. Genomic DNA was extracted and purified using QIAGEN DNeasy Tissue kit following the manufacture's protocol. Quantitative analysis by real-time PCR(qPCR) was performed in an ABI PRISM 7700 Sequence Detector (Applied Biosystems, Foster City, CA) using Platinum SYBR Green qPCR SuperMix and the following oligonucleotides: KrasBoth, Fw 5'- AGGCCTGCTGAAAATGACTG - 3', Rv 5' - TGGT TCCCTAACACCCAGTT – 3'; KrasWT, Fw 5' - GTGTTGGGATAGCTGTCGAC- 3', Rv 5' – GATATTCCGAATTCAGTGACTAC - 3'And *β-actin* as a reference gene, Fw 5' - CTTCTGCATCCTGTCAGCAA - 3', Rv 5' - GCTCTTTTCCAGCCTTCCTT - 3'. KrasBoth oligonucleotides were designed to detect both *Kras* mutant and wild-type alleles. Each amplification reaction was checked for the absence of nonspecific PCR products by running the dissociation protocol. Each run contained a standard curve of murine normal DNA derived from the pancreas of a wild-type mouse from 50 to 6.25 ng in triplicate, used as the control (with 2-copy of *Kras* alleles) for each target. The relative gene copy number for *Kras* mutant allele was calculated using the $2^{-\Delta\Delta Ct}$ method. $\Delta\Delta$Ct represents the difference between each cell line and the control (ΔCt of cell line with LOH - ΔCt of the control), with ΔCt being the average Ct for the target allele (mutant and wild-type or wild-type alone) minus the average Ct for the reference gene (*β-actin*). Samples were analyzed in triplicate. Because the wild-type *Kras* allele is absent in the cancer cell lines with LOH at *Kras*, as confirmed by using the KrasWT primers, values greater than 1.0 using the KrasBoth primers were considered positive for gene amplification. Similar protocol was used on the cDNA for the expression of *CXCL12*, *CXCR4, CSF-1,* and *CSCF1R* with using the same primers listed above for RT-PCR and actin-F: TCATCAGGTAGTCAGTGAGGTCGC; actin-R: ACCACACCTTCTACAATGAGCTG.

Mutational analyses of the *KRAS* gene. Genomic DNA (40ng per sample) was amplified with primers that had been designed to specifically amplify the codons 12, 13, and 61 of *KRAS*. The primers used are KRAS-E2-F-CGATACACGTCTGCAGTCAAC, KRAS-E2-R-ATTTACCTCTATTGTTGGATC, KRAS-E3-F-GTGCACTGTAATAATCCAGAC, and KRAS-E3-R-TAATTACTCCTTAATGTCAGC. Amplified products were purified and sequenced directly with either the forward or reverse PCR primers. Sequencing was performed by GENEWIZ, Inc (South Plainfield, NJ).

SNP analyses of human cancer cell lines. Genotypes were determined by the GeneChip® DNA Analysis Software Tool (GDAST, v3.0) using a 0.05 quality score setting genotypes were plotted with respect to their genomic position listed in the May 2004 assembly of the human genome. Four potential genotype calls were made: AA, BB, AB, and NoCall. No

distinction was made between homozygosity for either allele; each result was considered uninformative and provided only a visual reference to indicate genome coverage. Heterozygous calls indicated the retention of both alleles and were considered informative. Fractional allelic loss (FAL) based on SNP data was calculated for all chromosome arms as the percent of cell lines with partial or total allelic loss divided by the total number of cell lines analyzed.
