# Supplementary material for: Disruption of p16 and Activation of Kras in Pancreas Increase Ductal Adenocarcinoma Formation and Metastasis in vivo
Source: Oncotarget. 2011 Nov 23;2(11):862–73. doi: 10.18632/oncotarget.357 (PMC3259996; doi:10.18632/oncotarget.357)
Supplement: Supplementary file 3 [file oncotarget-02-862-s003.pdf]

SUPPLEMENTARY TABLE

Table S1. A representative histologic analysis of invasive pancreatic cancer in $p16^{-/-}$; *LSL-Kras*^{G12D}; *Pdx1-Cre* mice.

ID	Age (days)	Invasive CA* pattern	Invasive CA size	CA(%) sarcom*	CA(%) adeno*	CA(%) anapl*	METs**
8-289	42	diffuse	15mm	95	5		
7-59	74	diffuse	13mm	10	50	40	liver
7-992	83	focal	3mm	10	80	10	
7-883	84	focal	3 mm	20	5	75	
9-36	85	diffuse	30mm	40	10	50	LNs
7-478	88	diffuse	27mm		90	10	LNs
7-756	89	diffuse	16mm	10	80	10	LNs, liver
7-57	98	diffuse	14mm	5	75	20	liver
8-284	98	diffuse	20mm	80	15	5	
8-111	102	diffuse	20mm	20	60	20	
7-757	103	diffuse	20mm	20	80		
7-744	108	diffuse	20mm	70	20	10	liver, lung
8-99	109	diffuse	16mm	50	50		
7-56	110	diffuse	20mm	95	5		
8-285	112	diffuse	17mm	60	20	20	
8-810	116	diffuse	10mm	90	10		liver
8-490	121	diffuse	12mm	10	80	10	liver
8-901	122	multifocal	1- 5 mm	40	50	10	
7-513	123	diffuse	20mm	90		10	
7-565	128	diffuse	10mm	20	80		
8-282	136	diffuse	13mm	40	50	10	
7-65	141	diffuse	15mm	10	90		liver

*CA-cancer; sarcom-sarcomatoid; adeno-adenocarcinoma; anapl-anaplastic; METs-metastasis.
**Mice were sacrificed based on age for analysis of their primary pancreatic tumors, therefore not all of them had reached non-thriving stage and metastasis had not yet developed in some of them.

Table S2. Histologic analysis of pancreatic neoplasms in *p16$^{flox/flox}$*; *LSL- Kras^{G12D}*; *Pdx1-Cre* mice.

ID	Age (days)	Preinvasive neoplasm+	Invasive CA* pattern+	Invasive CA size	CA(%) sarcom	CA(%) adeno	CA(%) anapl.	METs**
11-257	49	PanIN-3	-					
18-128	88	PanIN-1	-					
18-140	88	PanIN-1	-					
19-268	93	PanIN-2	-					
20-857	93		diffuse	30mm	10	90		liver
20-792	98		diffuse	18mm	20	80		liver
20-622	98		diffuse	8 mm		80	20	
19-713	108	PanIN-1	-					
19-799	108	PanIN-3	-					
20-301	113		focal	3 mm		40	60	
18-717	120		diffuse	10 mm	80	10	10	
17-877	120		diffuse	30 mm	20	10	70	duo.
17-879	123		diffuse	28 mm		80	20	liver
17-758	128		diffuse	9 mm	75	15	10	duo.
17-855	128	PanIN-3	-					
16-104	136	PanIN-3	-					
15-269	146		diffuse	14 mm	5	80	15	liver

+The highest PanIN lesion was recorded if no invasive cancer was detected.
*CA-cancer; sarcom-sarcomatoid; adeno-adenocarcinoma; anapl-anaplastic; METs-metastasis.
**Mice were sacrificed based on age for analysis of their primary pancreatic tumors, therefore not all of them had reached non-thriving stage and metastasis had not yet developed in some of them.

Table S3. Two primary pancreatic cancer cell lines with LOH at *Kras* were analyzed by qPCR (SYBR Green) for *Kras* amplificaiton.

**Sample**	**ΔΔCt***	**Rel. copy number *Kras* mutant****	***Kras* amplification**
LOH at *Kras* #1	0.9233	1.0544	Negative
LOH at *Kras* #2	1.1633	0.8928	Negative

*Values provided represent means derived from triplicate assays of the same sample. Mean ΔΔCt values of *the cell line with LOH vs.* 2 copy control obtained from corresponding standard curves.

** Relative copy number values of *Kras* for each cell line with LOH.
